# A Phase Retrieval Method for 3D Shape Measurement of High-Reflectivity Surface Based on π Phase-Shifting Fringes

**DOI:** 10.3390/s23218848

**Published:** 2023-10-31

**Authors:** Yanjun Zhang, Junhua Sun

**Affiliations:** School of Instrumentation and Optoelectronic Engineering, Beihang University, Beijing 100191, China; mzbys@buaa.edu.cn

**Keywords:** fringe projection profilometry, overexposure, phase retrieval, 3D shape measurement

## Abstract

Fringe projection profilometry (FPP) has been widely used for 3D reconstruction, surface measurement, and reverse engineering. However, if the surface of an object has a high reflectivity, overexposure can easily occur. Image saturation caused by overexposure can lead to an incorrect intensity of the captured pattern images, resulting in phase and measurement errors of FPP. To address this issue, we propose a phase retrieval method for the 3D shape measurement of high-reflectivity surfaces based on π phase-shifting fringes. Our method only requires eight images to be projected, including three single-frequency three-step phase-shifting patterns and one pattern used to provide phase unwrapping constraints, called conventional patterns, as well as the π phase-shifting patterns corresponding to the four conventional patterns, called supplemental patterns. Saturated pixels of conventional fringes are replaced by unsaturated pixels in supplemental fringes to suppress phase retrieval errors. We analyzed all 16 replacement cases of fringe patterns and provided calculation methods for unwrapped phases. The main advantages of our method are as follows: (1) By combining the advantages of the stereo phase unwrapping (SPU) algorithm, the number of projected fringes is reduced. (2) By utilizing the phase unwrapping constraint provided by the fourth fringe pattern, the accuracy of SPU is improved. For highly reflective surfaces, the experimental results demonstrate the performance of the proposed method.

## 1. Introduction

**Figure 1:**
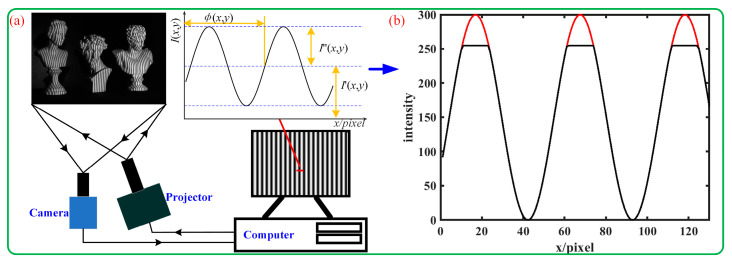
(**a**) Typical measurement system; (**b**) truncated fringe pattern intensity distribution (flat area) due to image saturation.

Over recent years, measuring the shape of three-dimensional (3D) objects has garnered significant interest among scholars and has been widely used in fields like industrial design, reverse engineering, and quality assessment [1,2]. In contrast, fringe projection profilometry (FPP) has attracted widespread interest among optical methods because of its benefits such as being non-contact, offering full-field inspection, and providing high resolution and precision [3,4,5,6]. As illustrated in Figure 1a, the FPP measurement system commonly consists of a projector and a camera. In FPP, 3D reconstruction is performed as follows [7]: The fringe pattern is projected onto the object surface, and an image of the fringe pattern deformed by the object surface is captured by a camera. From the captured images, a phase map based on the pixel information is then calculated. Finally, by using a calibrated phase-to-height mapping model or a binocular vision measurement model based on the desired measurement volume, the 3D coordinates of the surface of the object from the phase map can be derived.

In practice, FPP usually assumes that the surface of the object has diffuse reflection or close to diffuse reflection. Therefore, when the light intensity surpasses the camera sensor’s capture threshold because of extreme brightness, the actual intensity is truncated to the camera sensor’s highest quantization level, so it always causes the camera sensor to saturate. Assuming the camera is an 8-bit camera, if the image intensity value is higher than 255, it will saturate, as shown in Figure 1b. This means that the fringe pattern of highlight modulation cannot be decoded correctly, leading to significant measurement errors in the highlight areas. Currently, in industrial applications, a common solution to address this issue involves spraying a thin powder layer on the object to ensure a diffused surface before measurement. This additional step, however, is tedious and prolongs the process since the object must be cleaned afterward. Moreover, the ultimate precision often depends on the uniformity and thickness of the applied powder [8].

To tackle saturation-induced phase mistakes, several innovative techniques have been introduced. They generally fall into three types: exposure-based methods, projection-based methods, and other algorithms. The exposure-based method is to fuse images acquired at different exposure times into one image to avoid image saturation. Zhang et al. [9] proposed a high-dynamic-range scanning technique designed to handle differences in the reflectivity of different surfaces. The method merges images with varied exposures into a collection of phase-shifting images, choosing the most luminous untruncated intensity for every pixel. By replacing pixels saturated at high exposure with corresponding pixels at low exposure, saturated areas can be correctly measured without affecting other areas. Jiang et al. [10] introduced a technique for creating composite fringe images by modifying the camera’s exposure time and the light intensity of the projected fringe. The method selects pixels based on the highest modulation intensity to minimize ambient light effects and automatically select parameters. However, its application is intricate, and it necessitates at least a fivefold increase in fringe images compared to conventional phase assessment. Feng et al. [11] divided the measured surface reflectance into several groups and then adaptively predicted the optimal exposure time for each one. This approach effectively addresses both bright and dark areas on the test surface. Using these optimal exposure times, the original pattern image is captured and then used to synthesize an HDR image. However, estimating the camera response function using the histogram of the sequence of images leads to blocking artifacts and is not intelligent enough to choose the predicted exposure time. Ekstrand et al. [12] proposed an automatic exposure technology that can automatically predict the required exposure time according to the reflectivity of the surface of the measured object. This approach minimizes manual input and enhances the 3D measurement system’s intelligence. Still, since the predicted exposure time is based on the object’s brightest area, it often does not suit the needs of darker regions within the same measurement context. Liu et al. [13] introduced a method to use LRR to process object surfaces through a dual-camera structure light system. This method needs to project 256 uniformly increasing grayscale white images on the object under the test to create a mask image, and the whole process is very time-consuming.

Projection-based methods prevent intensity saturation by adjusting the intensity and contrast of projected fringe patterns. Waddington and Kofman [14] proposed a technique that automatically adjusts the intensity of projected pattern patterns to adapt the maximum input grayscale level to ambient light, to avoid saturation. This method merges raw fringe pattern images taken from various positions, yet it may take more time to complete than the multiple-exposure approach. Li et al. [15] proposed a method. First, a sinusoidal image with high gray levels is used to determine the saturated pixels of highly reflective surfaces. Then, the correlation between the projector and camera images at the saturated pixel is established by gathering low-grayscale sinusoidal images and solving their phases. Next, the correlation between the grayscale intensity of a captured image and the grayscale of the projected image is determined, and the projected grayscale of the saturated area as a whole is adjusted to avoid imaging saturation. Lin et al. [16] proposed a pixel-level adaptive fringe projection method. By projecting a sinusoidal fringe image with a high gray scale, the saturated areas of highly reflective surfaces are identified, and contours are extracted. Using the absolute phase and coordinate correspondence of contour pixels, the correspondence between camera pixels and projected pixels is obtained. By projecting multiple uniform grayscale images and collecting images, the optimal projection intensity of each pixel is calculated to achieve pixel-level adaptive adjustment. Chen et al. [17] suggested an adaptive method for fringe projection that simplifies the computational procedure. The contour tracking algorithm determines the contour of the saturated area, and it establishes the mapping relationship between the grayscale of the pixel on the contour and the grayscale of the projected pixel, simplifying the experimental process and reducing the projection of low-grayscale fringes. However, this method approximates the reflection characteristics of pixels, and the adjustment of the projected image is overall regional, which cannot achieve precise adjustment at the pixel level. Xu et al. [18] introduced a novel AFP measurement technique for speckle image pixel matching. Firstly, the adaptive projection intensity for the two grayscale modes is computed, and then speckle patterns are projected to match captured images and projected patterns. Finally, adaptive patterns are generated. Just three more patterns need to be projected to measure HDR surfaces, which significantly improves efficiency.

In addition, researchers have proposed other methods for measuring high-reflectivity surfaces, such as multi-camera observation [19,20], color filters [21], polarizing filters [22,23], photometric stereo [24], and post-processing compensation [25,26,27].

In short, exposure-based methods require the number of patterns to be *N* times that of normal patterns (generally *N* ≥ 3), and the exposure time needs to be based on empirical values. Projection-based methods need to adjust the projection fringe according to the different optical conditions of the surface of the object to be measured. The process is relatively complicated, and it is only applicable to a single viewing angle and a single ambient light. If the position of the measured object changes relative to the measurement system, the patterns need to be re-programmed; in addition, the programming of the projected patterns is time-consuming and difficult to use in practice. Other methods require additional hardware or system configurations or increase complexity in image mask processing and registration to acquire high-quality images for 3D reconstruction.

For flexible phase solving and 3D reconstruction of overexposed surfaces, Jiang et al. [28] introduced an HDR 3D scanning approach using extra supplemental fringe patterns. The approach revolves around using both the original and supplemental pattern images, where the supplemental pattern compensates for the highlighted pixel intensities in the original pattern. Yet, Jiang executed phase unwrapping either by the spatial domain method or the Gray code technique. As we all know, the spatial domain method cannot measure isolated objects. The Gray code combined with the phase-shifting method requires a large number of fringes. Assuming that the sinusoidal fringes projected by the phase-shifting method have 73 periods, ceil(log_2_73) = 7 Gray code patterns are required. Including supplemental patterns, a total of 2 × (7 + 3) = 20 patterns are required. If the traditional three-frequency three-step phase-shifting method is used, at least 2 × (3 + 3 + 3) = 18 images are required for phase unwrapping. To decrease the pattern count and maintain the ability to measure isolated objects, we integrate the stereo phase unwrapping (SPU) method [29,30] for phase extraction. However, SPU requires the number of frequencies generally to not exceed 30, which limits the accuracy of 3D reconstruction [31]. By integrating SPU with Jiang’s idea, the accuracy of phase extraction and 3D reconstruction in the overexposed region is restricted.

To minimize the necessary pattern count and enhance phase extraction quality in the highly reflective regions of an object without compromising precision, we propose a phase retrieval method for highly reflective 3D shape measurement based on π phase-shifting fringe patterns. Firstly, according to the internal constraints of four patterns, the candidate fringe order values (no more than 30) are obtained; on this basis, the final fringe order is determined in combination with the 3D geometry constraints, to obtain the absolute phase without entanglement. In this process, phases are classified and retrieved according to 16 different exposure conditions, and the saturated pixels of the conventional fringes are replaced by the unsaturated pixels in the supplemental fringes, to improve the accuracy and completeness of the highly reflective areas.

The remainder of this paper is organized as follows: Section 2 explains the principle of the proposed method. Section 3 presents some simulation and experimental results related to the proposed method. Section 4 concludes the paper.

## 2. Principle

### 2.1. Principle of Phase-Shifting and Phase-Coding Method

Among the different FPP techniques, the phase-shifting technique provides high-quality phase extraction through a set of phase-shifting fringe images. For *N*-step phase-shifting, each fringe image $I_{n}(x,y)$ can be expressed as follows:

(1) $$I_{n}(x,y)=A(x,y)+B(x,y)\cos\left[\phi (x,y)+2n\pi /N\right]$$

where A(x,y) represents the ambient light, B(x,y) represents the intensity modulation, and ϕ(x,y) is the phase. To calculate the phase ϕ(x,y), the least-squares method can be used to solve the over-constrained simultaneous equations when *N* is greater than or equal to 3.

(2) $$\varphi (x,y)=\tan^{-1}\left(\frac{\sum_{n=1}^{N}I_{n}(x,y)\sin(2n\pi /N)}{\sum_{n=1}^{N}I_{n}(x,y)\cos(2n\pi /N)}\right)$$

In Equation (2), φ(x,y) is in the range (−π, π) due to the arctangent operation. To obtain an absolute phase map without 2π discontinuity, it is necessary to add an integer multiple of k(x,y) to the discontinuous phase [32], as shown in the following formula:

(3) ϕ(x,y)=φ(x,y)+k(x,y)×2π

The phase-encoding method proposed by Wang et al. [33] uses a stair function to embed the codeword into the phase to obtain the initial phase, as shown in the following formula:

(4) $$\phi^{s}(x,y)=-\pi +[x/P]\times 2\pi /N$$

where N is the number of fringe frequencies, [x/P]=k(∈[1,N]) is the fringe order, and P is the number of pixels per period.

Then, the initial phase is substituted into the n-step phase-shift fringes according to the following formula:

(5) $$I_{n}^{s}(x,y)=A(x,y)+B(x,y)\cos\left[\phi^{s}(x,y)+2n\pi /N\right]$$

The phase-encoding fringe pattern is then projected onto the object using a DLP projector and then captured using a CCD camera. The stair phase is obtained through the inverse solution of the captured image.

(6) $$\phi^{s}(x,y)=\tan^{-1}\left[\frac{\sum_{n=1}^{n}I_{n}^{s}(x,y)\sin(2\pi /n)}{\sum_{n=1}^{n}I_{n}^{s}(x,y)\cos(2\pi /n)}\right]$$

Then, the stair phase is used to calculate the fringe order according to the following formula:

(7) $$k(x,y)=\mathrm{Round}\left[N\left(\phi^{s}(x,y)+\pi \right)/2\pi \right]$$

Finally, the fringe order is used to convert the wrapped phase into an absolute phase using Equation (3).

### 2.2. The Proposed Algorithm Principle

We use four conventional fringes combined with the 3D geometry constraint method to solve the phase under normal exposure conditions, and for each different overexposure case, we use the method of combining four supplemental fringes to deal with it. Firstly, different exposure situations are classified, and then the corresponding wrapped phase and unwrapped phase calculation methods are described.

#### 2.2.1. Classification of Different Exposure Cases

The four conventional fringes projected include a three-step phase-shifting fringe with a phase shifting of 2π/3 and a sinusoidal fringe used to provide order constraints for the phase unwrapping. The captured images are represented as

(8) $$\left\{\begin{array}{l} I_{1}^{con}(x,y)=A(x,y)+B(x,y)\cos[\phi (x,y)-2\pi /3]\\ I_{2}^{con}(x,y)=A(x,y)+B(x,y)\cos[\phi (x,y)]\\ I_{3}^{con}(x,y)=A(x,y)+B(x,y)\cos[\phi (x,y)+2\pi /3]\\ I_{4}^{con}(x,y)=A(x,y)+B(x,y)\cos[\phi (x,y)-C_{s}(x,y)] \end{array}\right.$$

where $C_{s}(x,y)$ encodes the fringe order and can also be written as follows:

(9) $$C_{s}(x,y)=-\pi +[x/P]\times 2\pi /N$$

The four supplemental fringes projected are obtained by shifting the phase π of the four conventional fringes, respectively. The captured images are represented as

(10) $$\left\{\begin{array}{l} I_{1}^{sup}(x,y)=A(x,y)+B(x,y)\cos[\phi (x,y)-2\pi /3+\pi ]\\ I_{2}^{sup}(x,y)=A(x,y)+B(x,y)\cos[\phi (x,y)+\pi ]\\ I_{3}^{sup}(x,y)=A(x,y)+B(x,y)\cos[\phi (x,y)+2\pi /3+\pi ]\\ I_{4}^{sup}(x,y)=A(x,y)+B(x,y)\cos[\phi (x,y)-C_{s}(x,y)+\pi ] \end{array}\right.$$

The different exposure cases of the proposed method are analyzed as follows: It is divided into one pattern overexposed, two patterns overexposed, three patterns overexposed, and four patterns overexposed based on the relationship between the pattern grayscale value and 255. Here, the overexposure conditions of different patterns and the corresponding usage patterns are listed in the following Table 1. There are 1 + 4 + 6 + 4 + 1 = 16 cases in total. Since the phase retrieval of the phase-shifting algorithm is a pixel-by-pixel operation, we drop the coordinate index (*x*, *y*) to simplify the notation.

For different exposure cases, we will describe the calculation of the texture, modulation, wrapped phase, and unwrapped phase below.

#### 2.2.2. Wrapped Phase, Texture, and Modulation Calculation

A(x,y) is often viewed as a texture image that can be used for visualization or provide clues for visual analysis. For areas that are in shadow, dark, or saturated, the camera’s captured image undergoes minimal modulation by the sinusoidal projection, bringing the modulation near zero. Hence, modulation is commonly employed as a filter. Areas with modulation values below or above set thresholds are typically disregarded in further analysis.

It should be noted that $I_{4}(I_{4}^{con}\;or\;I_{4}^{sup})$ does not participate in the calculation of the wrapped phase, so the case involving $I_{4}$ overexposure can be classified as the case containing only $I_{1}-I_{3}(I_{1}^{con}-I_{3}^{con}\;\mathrm{or}\;I_{1}^{sup}-I_{3}^{sup})$ overexposure. For example, the wrapped phase calculations in Case 2_4, Case 3_3, Case 3_5, Case 3_6, Case 4_2, Case 4_3, Case 4_4, and Case 5_1 are the same as those in Case 1_1, Case 2_1, Case 2_2, Case 2_3, Case 3_1, Case 3_2, Case 3_4, and Case 4_1, respectively. Therefore, the calculation of the wrapped phase can be divided into eight cases: Case 1_1, Case 2_1, Case 2_2, Case 2_3, Case 3_1, Case 3_2, Case 3_4, and Case 4_1. For the solution of the wrapped phase, we can refer to Jiang’s method: In the general scene, only the conventional fringe image is used to analyze and wrap the phase, and when one or two of the conventional fringe images $I_{1}^{con}(x,y)$, $I_{2}^{con}(x,y)$, and $I_{3}^{con}(x,y)$ are saturated, it is replaced with the corresponding $I_{1}^{sup}(x,y)$, $I_{2}^{sup}(x,y)$, and $I_{3}^{sup}(x,y)$; then, the simultaneous equations are solved to calculate the new wrapped phase. In particular, when the conventional fringe images are overexposed (including supplemental fringe images also being overexposed), all conventional fringe images and supplemental fringe images are used in the phase calculation in a least-squares manner to minimize the phase error caused by saturation.

But Jiang did not explain the texture and modulation solution. The calculation of texture and modulation is explained below. We can transform the three equations of these eight cases into the form of a system of linear equations ax=b, where a is the coefficient matrix, x is the variable vector, and b is the result vector. Taking Case 2_1 as an example, the specific solution process is as follows:

The expression of $I_{1}$, $I_{2}$, $I_{3}$ is rewritten as follows:

(11) $$\left\{\begin{array}{l} I_{1}=A-B\cos(\phi )\cos(2\pi /3)-B\sin(\phi )\sin(2\pi /3)\\ I_{2}=A+B\cos(\phi )\\ I_{3}=A+B\cos(\phi )\cos(2\pi /3)-B\sin(\phi )\sin(2\pi /3) \end{array}\right.$$

Then we can write a, b, x as follows:

(12) $$a=\left[\begin{array}{ccc} 1 & -\cos(2\pi /3) & \sin(2\pi /3)\\ 1 & 1 & 0\\ 1 & \cos(2\pi /3) & \sin(2\pi /3) \end{array}\right]$$

(13) $$x=\left[A;B\sin(\phi );B\cos(\phi )\right]$$

(14) $$b=\left[I_{1};I_{2};I_{3}\right]$$

A system of linear equations is solved to obtain A, Bsin(ϕ), and Bcos(ϕ). Finally, we can solve for B with $B=\sqrt{{\left(B\sin(\phi )\right)}^{2}+{\left(B\cos(\phi )\right)}^{2}}$.

Similarly, we can list $a_{i}$ for the rest of the cases.

$$a_{2}=\left[\begin{array}{ccc} 1 & \cos(2\pi /3) & -\sin(2\pi /3)\\ 1 & -1 & 0\\ 1 & \cos(2\pi /3) & \sin(2\pi /3) \end{array}\right],\;a_{3}=\left[\begin{array}{ccc} 1 & \cos(2\pi /3) & \sin(2\pi /3)\\ 1 & 1 & 0\\ 1 & -\cos(2\pi /3) & \sin(2\pi /3) \end{array}\right],$$

$$a_{12}=\left[\begin{array}{ccc} 1 & -\cos(2\pi /3) & \sin(2\pi /3)\\ 1 & -1 & 0\\ 1 & \cos(2\pi /3) & \sin(2\pi /3) \end{array}\right],\;a_{13}=\left[\begin{array}{ccc} 1 & -\cos(2\pi /3) & -\sin(2\pi /3)\\ 1 & 1 & 0\\ 1 & -\cos(2\pi /3) & \sin(2\pi /3) \end{array}\right]$$

(15) $$a_{23}=\left[\begin{array}{ccc} 1 & \cos(2\pi /3) & \sin(2\pi /3)\\ 1 & -1 & 0\\ 1 & -\cos(2\pi /3) & \sin(2\pi /3) \end{array}\right],\;a_{123}=\left[\begin{array}{ccc} 1 & -\cos(2\pi /3) & -\sin(2\pi /3)\\ 1 & -1 & 0\\ 1 & -\cos(2\pi /3) & \sin(2\pi /3) \end{array}\right]$$

By the same method, all the A and B values can be determined. Once the value of B for each pixel is obtained, we can identify valid points using the following equation:

(16) Mask(x,y)=B(x,y)>ThrVal

where ThrVal is the modulation threshold value.

#### 2.2.3. Unwrapped Phase Calculation

According to the obtained wrapped phase and the constraints satisfied by the fringe order, the phase unwrapping can be further combined with the geometry constraints of the measuring system. Fortunately, all eight combinations of $I_{4}^{con}$ and $I_{4}^{sup}$ with $I_{1}-I_{3}(I_{1}^{con}-I_{3}^{con}\;\mathrm{or}\;I_{1}^{sup}-I_{3}^{sup})$ provide initial constraints on the phase unwrapping. The specific analysis is as follows:

Since $C_{s}(x,y)$ in Equation (8) encodes the fringe order k(x,y), it can be used to obtain the unwrapped phase. Expanding $I_{4}^{con}(x,y)$ in Equation (8) gives:

(17) $$\begin{array}{ll} I_{4}^{con}(x,y) & =A(x,y)+B(x,y)\cos[\phi (x,y)+C_{s}(x,y)]\\ & =A(x,y)+B(x,y)\cos[\phi (x,y)]*\cos[C_{s}(x,y)]-B(x,y)\sin[\phi (x,y)]*\sin[C_{s}(x,y)] \end{array}$$

where A(x,y) can be calculated from the first three images in Equation (8) and can be expressed as

(18) $$A=(I_{1}^{con}+I_{2}^{con}+I_{3}^{con})/3$$

Bcos(ϕ) is calculated as follows:

(19) $$\left\{\begin{array}{l} 2I_{2}^{con}=2A+2B\cos(\phi )\\ I_{1}^{con}+I_{3}^{con}=2A+2B\cos(\phi )\cos(2\pi /3) \end{array}\right.$$

(20) $$\Rightarrow I_{1}^{con}+I_{3}^{con}-2I_{2}^{con}=-3B\cos(\phi )$$

(21) $$\Rightarrow B\cos(\phi )=-(I_{1}^{con}+I_{3}^{con}-2I_{2}^{con})/3$$

Bsin(ϕ) is calculated as follows:

(22) $$I_{1}^{con}-I_{3}^{con}=2B\sin(\phi )\sin(2\pi /3)=\sqrt{3}B\sin(\phi )$$

(23) $$\Rightarrow B\sin(\phi )=(I_{1}^{con}-I_{3}^{con})/\sqrt{3}$$

Substituting Equations (18), (21), and (23) into Equation (17) gives

(24) $$-\frac{\left(I_{1}^{con}-I_{1}^{con}\right)}{\sqrt{3}}\sin C_{s}-\frac{\left(I_{1}^{con}+I_{3}^{con}-2I_{2}^{con}\right)}{3}\cos C_{s}-\frac{\left(I_{1}^{con}+I_{2}^{con}+I_{3}^{con}\right)}{3}+I_{4}^{con}=0$$

**Figure 2:**
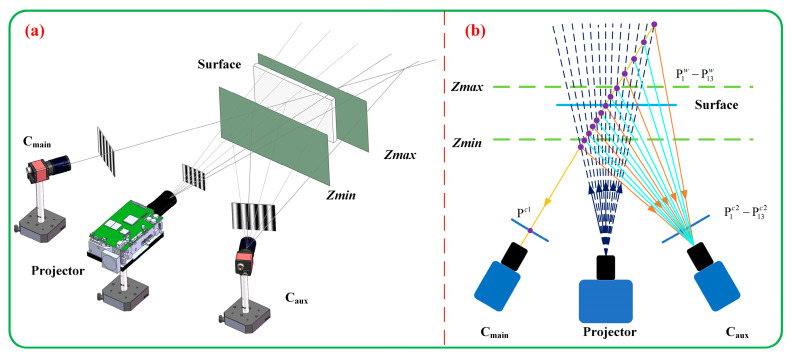
Schematic diagram of SPU. (**a**) System composition. (**b**) Different frequencies.

So, after $I_{1}^{con}(x,y)$, $I_{2}^{con}(x,y)$, $I_{3}^{con}(x,y)$, $I_{4}^{con}(x,y)$ of a point are obtained, the corresponding $C_{s}(x,y)$ can be solved using Equation (24). Given that Equation (24) comprises sine and cosine functions, obtaining $C_{s}(x,y)$ directly can be difficult. Since k(x,y)(∈[1,N]) is an integer, we obtain *N* values of $C_{s}(x,y)$. Then, we substitute the *N* candidate values of $C_{s}(x,y)$ into the left side of Equation (24) to obtain *N* values. We can choose a value of $C_{s}(x,y)$ that corresponds to the minimum value of Equation (24) to solve for the fringe order. However, this becomes difficult due to the presence of noise. Therefore, we select Q points near the minimum value (after experimental verification, generally select about 15 points). In this way, the original candidate points are reduced from *N* (73 is used in this paper) to Q, and then the SPU is used to determine the final fringe order. The detailed process is as follows:

The system layout is depicted in Figure 2a, with C_main_ acting as the main camera and C_aux_ serving as an auxiliary camera to aid C_main_ in determining the unwrapped phase. Suppose $p^{cm}(x^{cm},y^{cm})$ represents a pixel of C_main_. Its correspondence in the projector is $y^{p}=\phi^{cm}(x^{cm},y^{cm})/(2*\pi )$. $\varphi^{cm}(x^{cm},y^{cm})$ can be determined by the three-step phase-shifting algorithm. Solely from $\varphi^{cm}(x^{cm},y^{cm})$, pinpointing the precise corresponding point in world coordinates is not feasible. But we can estimate all potential 3D points $P_{Q}^{w}\left(X^{w},Y^{w},Z^{w}\right)$ using the unwrapped phases obtained for different fringe patterns; the calculation formula is as follows:

(25) $$\left(\begin{array}{c} X^{w}\\ Y^{w}\\ Z^{w} \end{array}\right)={\left(\begin{array}{c} m_{11}^{cm}-m_{31}^{cm}x^{cm},m_{12}^{cm}-m_{32}^{cm}x^{cm},m_{13}^{cm}-m_{33}^{cm}x^{c1}\\ m_{21}^{cm}-m_{31}^{cm}y^{cm},m_{22}^{cm}-m_{32}^{cm}y^{cm},m_{23}^{cm}-m_{33}^{cm}y^{c1}\\ m_{21}^{p}-m_{31}^{p}y^{p},m_{22}^{p}-m_{32}^{p}y^{p},m_{23}^{p}-m_{33}^{p}y^{p} \end{array}\right)}^{-1}\left(\begin{array}{c} m_{14}^{cm}-m_{34}^{cm}x^{cm}\\ m_{24}^{cm}-m_{34}^{cm}y^{cm}\\ m_{24}^{p}-m_{34}^{p}y^{p} \end{array}\right)$$

where $\left(\begin{array}{cccc} m_{11}^{p} & m_{12}^{p} & m_{13}^{p} & m_{14}^{p}\\ m_{21}^{p} & m_{22}^{p} & m_{23}^{p} & m_{24}^{p}\\ m_{31}^{p} & m_{32}^{p} & m_{33}^{p} & m_{34}^{p} \end{array}\right)=M^{p}$ and $\left(\begin{array}{cccc} m_{11}^{cm} & m_{12}^{cm} & m_{13}^{cm} & m_{14}^{cm}\\ m_{21}^{cm} & m_{22}^{cm} & m_{23}^{cm} & m_{24}^{cm}\\ m_{31}^{cm} & m_{32}^{cm} & m_{33}^{cm} & m_{34}^{cm} \end{array}\right)=M^{cm}$. $M^{p}$ and $M^{cm}$ are the parameters of the projector and C_main_, respectively, which are known after system calibration. The parameters in Equation (25) can be implemented using a lookup table indexed $\left(x^{c},y^{c}\right)$ (camera column and row indices), reducing the overall computational complexity associated with exporting a 3D point cloud [34].

These 3D points $P_{Q}^{w}\left(X^{w},Y^{w},Z^{w}\right)$ sharing the same wrapped phase but differing in fringe sequence are mapped to the plane of C_aux_ to obtain a set of 2D candidate points $P_{{}^{Q}}^{c2}$. In C_aux_, we look for the matching pixels of $P_{{}^{q}}^{c2}(q\in [1,Q])$. Since they should have similar properties, we consider $P^{c1}$ and $P_{q}^{c2}$ having the most similar wrapped phase as correctly matched. Consequently, C_main_’s unwrapped phase can be expressed as

(26) $$\phi^{cm}(x^{cm},y^{cm})=\varphi^{cm}(x^{cm},y^{cm})+k(x^{cm},y^{cm})\cdot 2\pi ,k(x^{cm},y^{cm})\in [k_{1},k_{2},\ldots k_{Q}]$$

where $\phi^{cm}\left(x^{cm},y^{cm}\right)$ is the wrapped phase of C_main_, $k\left(x^{cm},y^{cm}\right)$ is the fringe order, and $k_{1},k_{2},\ldots k_{Q}$ indicate the candidate fringe orders.

It should be pointed out that the reason why SPU is not used directly is that employing higher-frequency fringe patterns results in precise phases, making it preferable to use a higher frequency for high-precision 3D shape assessment. Yet, as illustrated by the orange lines of C_aux_ in Figure 2b, projecting in high-frequency modes leads to an excess of potential candidates in the measuring scope, potentially causing phase confusion. Certainly, by utilizing *Z_min_* and *Z_max_*, we can further constrict the measurement range. In this manner, it is challenging for us to confirm that the measured object remains within such a narrow range, and this difficulty is amplified especially when the object is in motion. After the phase unwrapping constraints are provided by the fourth fringe, we can reduce the number of candidate points for the fringe order to about 10. At this time, the reliability of determining the fringe order by using SPU is greatly enhanced, as shown in all lines of C_aux_ in Figure 2b. At the same time, the measurement accuracy is maintained because the high-frequency fringes are still used.

In conclusion, we use the geometric constraints of the system combined with the phase unwrapping constraint to overcome the dilemma of frequency selection and the robustness of the phase unwrapping to improve the performance of the phase unwrapping. In practical measurements, one must consider the potential inaccuracies arising from suboptimal system calibration. Additionally, the geometric constraints alone are not sufficient. To enhance accuracy, methods like left–right consistency verification [35] and edge point refinement [29] are incorporated to effectively discard any erroneous candidates.

Jiang did not explain how to deal with overexposure when using Gray codes to obtain fringe orders. Here, we explain the strategy for dealing with overexposure in the proposed phase unwrapping method. If it is considered that the fringe may be overexposed, the fringe corresponds to a different phase unwrapping formula. Fortunately, phases in all cases can be unwrapped by this method. Taking case 2 as an example, after Equation (8) becomes $I_{4}^{con}(x,y)=A(x,y)-B(x,y)\cos[\varphi (x,y)+C_{s}(x,y)]$, it can be expanded into the relationship of A, Bcos(φ), and Bsin(φ), so an equation similar to Equation (24) can be obtained, and the unwrapped phase can still be obtained by the same method. The process will not be repeated, and the specific calculation formula is shown in Table 2.

## 3. Experiments

### 3.1. Simulation Experiments

Using the proposed method, simulations were carried out for cases of fringes with normal exposure and overexposure.

#### 3.1.1. Normal Exposure Scene

**Figure 3:**
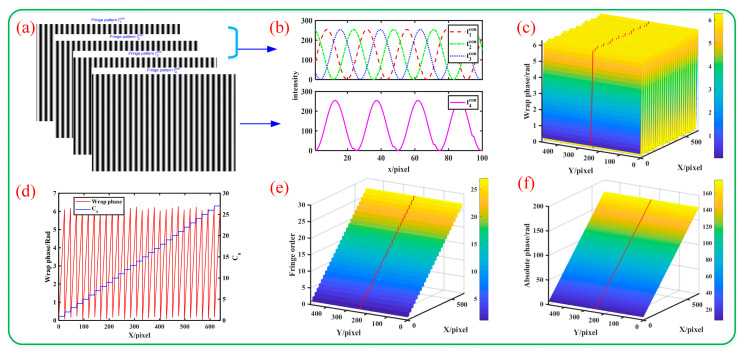
Simulation process: (**a**) pattern image; (**b**) grayscale distribution; (**c**) wrapped phase map; (**d**) wrapped phase and distribution; (**e**) fringe order map; (**f**) unwrapped phase map. Note: the red line in subfigures (**c**,**e**,**f**) represents the change trend of a certain section.

To simulate the normal exposure scene, we set the amplitude *A* and modulation *B* as A=B=127.5. Four sinusoidal fringes are generated, as shown in Figure 3a. The grayscale distribution of a row is shown in Figure 3b. At this time, the range of the image gray value range is [0,255], and there will be no overexposure. The wrapped phase and unwrapped phase calculated according to the proposed algorithm are shown in Figure 3c–f. Figure 3c represents the calculated wrapped phase, Figure 3d is the wrapped phase distribution map of a row in Figure 3c, Figure 3e is the calculated fringe order, and Figure 3f is the calculated unwrapped phase map. It can be seen that the calculated unwrapped phase is a smooth curved surface, and the phase distribution of a row is a smooth straight line.

#### 3.1.2. Overexposure Scene

**Figure 4:**
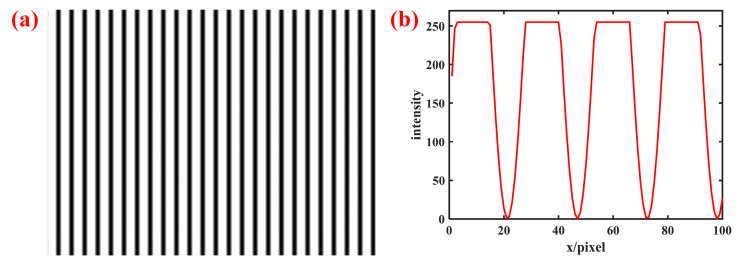
Schematic diagram including overexposed areas: areas larger than 255 will be truncated, resulting in phase solution errors. (**a**) Fringe pattern; (**b**) grayscale distribution of a certain row of the fringe pattern.

In order to simulate the overexposure scene, the amplitude *A* and modulation *B* are set to A=B=200, and the range of the image grayscale value is [0,400], so there will be some values greater than 255, which means overexposure occurs. Eight sinusoidal fringes are generated, one of which is shown in Figure 4a. The part of the overexposed three-step phase-shifting curve that exceeds 255 is truncated; that is, all values are limited, as shown in Figure 4b; this will cause the curve to lose a lot of information, which will affect the subsequent phase unwrapping.

**Figure 5:**
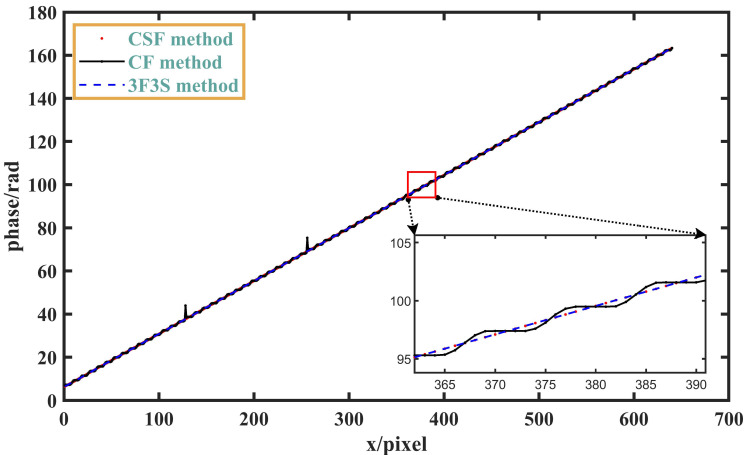
Phase distribution of different methods (*A* = *B* = 200): CSF method represents our method of combining conventional and supplemental patterns; CF method represents our method using conventional patterns only; 3F3S method represents the three-frequency heterodyne three-step phase-shifting method.

The normal three-step phase-shifting fringes are first taken as input, and the wrapped phase and absolute phase are resolved. Since there is no noise interference, the obtained absolute phase can be regarded as the true value. The visualized result of the absolute phase curve is shown as the blue point in Figure 5, and it can be seen that the absolute phase curve in the figure is a smooth straight line.

Then, the overexposed three-step phase-shifting fringes are used as input, and the absolute phase is analyzed according to the proposed algorithm without supplemental fringes. The obtained visualization result of the absolute phase curve is shown as the black dot in Figure 5, and it can be seen that the phase curve has some fluctuations due to the existence of overexposure.

Next, the proposed algorithm combined with supplemental fringes is used for phase analysis, and the obtained absolute phase curve is shown in the red line in Figure 5. It can be seen that the analyzed absolute phase curve is a smooth straight line, and the complete original information is restored.

**Figure 6:**
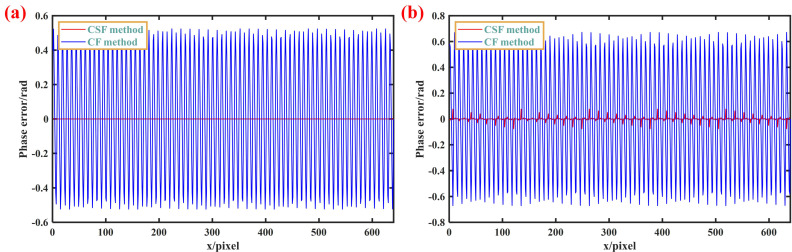
Phase error distribution curves of different methods. (**a**) *A* = *B* = 255; (**b**) *A* = *B* = 300.

**Figure 7:**
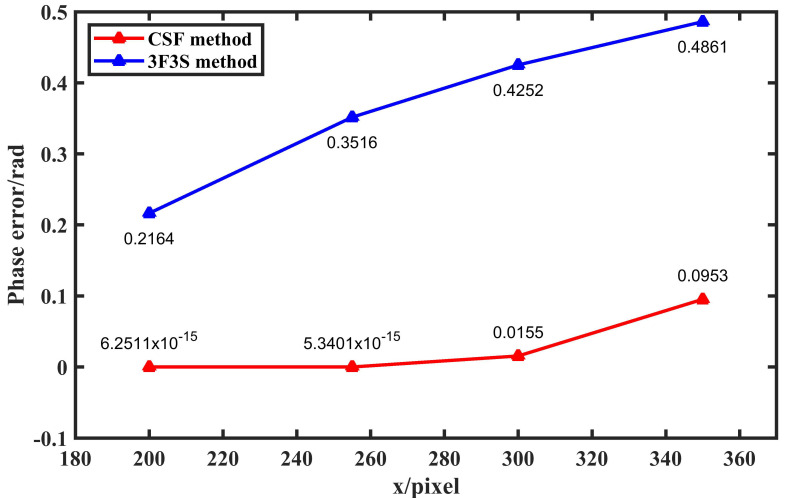
Phase error distribution.

To further quantitatively analyze the performance of the algorithm under different overexposure conditions, *A* and *B* are assigned values of 200, 255, 300, and 350, and the corresponding phase errors are calculated. The phase error curves at *A* = *B* = 255 and *A* = *B* = 300 are shown in Figure 6, and the error RMS in the four cases is shown in Figure 7. It can be seen that within twice the dynamic range, the phase error of this method is very small, at the level of 10^−15^. Even when twice the dynamic range is exceeded, our method is still better than the traditional method.

**Figure 8:**
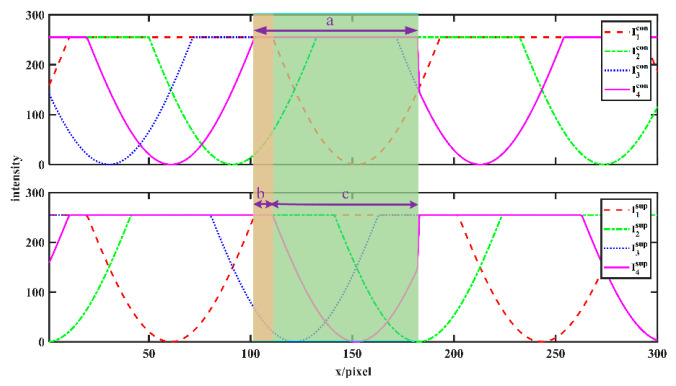
The distribution of gray values for a certain row. Area a is the overexposed area of the fourth conventional pattern, and the corresponding areas b and c are the overexposed area and normally exposed area of the supplemental pattern, respectively.

Jiang did not mention the quantitative analysis of improving the dynamic range; we will conduct a simple analysis here. To simulate an overexposed scene, we set *A* = *B* = 300. The distribution of gray values for a certain row of all eight images (conventional and supplemental fringes) is shown in Figure 8.

**Figure 9:**
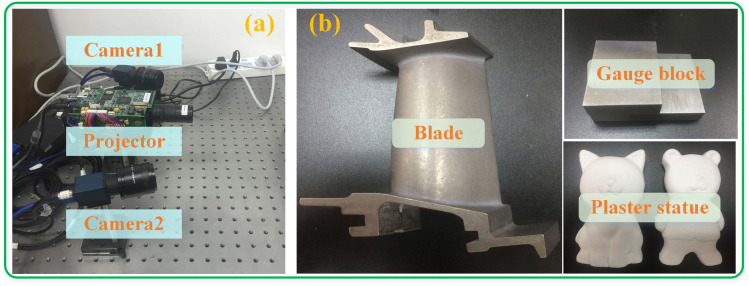
Experimental system and objects to be tested. (**a**) Experimental system; (**b**) objects to be tested, including gauge blocks to verify accuracy and plaster statues and a blade to verify measurement performance in highly reflective areas.

It should be noted that when A=B∈[0,127.5], $I^{con}=A+B*\cos\theta \in [0,255]$ and $I^{sup}=A-B*\cos\theta \in [0,255]$; that is, the conventional patterns and supplemental patterns will not be overexposed at this time. When A=B∈[0,255], $I^{con}=A+B*\cos\theta \in [255,510]$ and $I^{sup}=A-B*\cos\theta \in [0,255]$; that is, $I^{sup}$ can still be used instead of $I^{con}$ for phase calculation at this time. When the ranges of *A* and *B* are larger, there will be a situation where both the conventional pattern $I^{con}$ and the supplemental pattern $I^{sup}$ are overexposed. As shown in Figure 9, area a is the overexposed area of the fourth conventional pattern, and the corresponding area c is the normally exposed area of the supplemental pattern. However, the corresponding area b is the overexposed area of its supplemental pattern. In other words, our method doubles the dynamic range. But even if both the conventional patterns and supplemental patterns are overexposed, the phase error is still smaller than that of the conventional method, as shown in Figure 8.

### 3.2. Physical Experiments

The binocular measurement system consisted of two MER-504-10GM-P Daheng industrial cameras (resolution 2448 × 2048) and a DLP Light Crafter 4500 TI projector (resolution 912 × 1140). As shown in Figure 9a. The camera was synchronized by the trigger signal of the projector. The measured objects were metal gauge blocks, plaster statues, and aero-engine turbine blades, as shown in Figure 9b. The accuracy of the method proposed in this paper and its performance in the measurement of highly reflective scenes were verified by the experimental system built.

#### 3.2.1. Accuracy Verification

**Figure 10:**
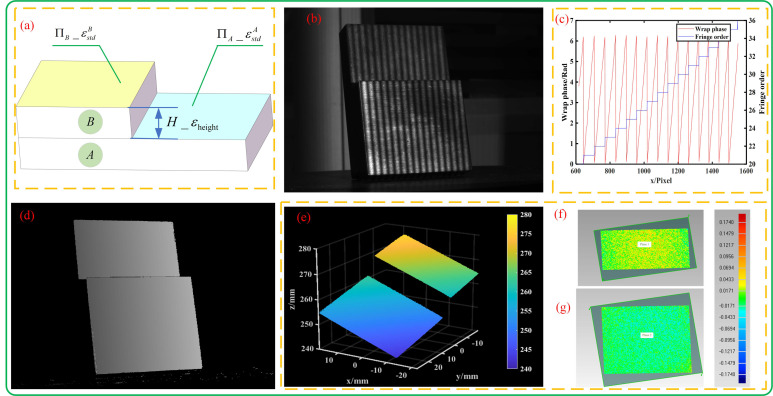
Stepped block measurement under normal exposure: (**a**) schematic diagram of the stepped block used to evaluate the measurement accuracy; (**b**) the fringe pattern of the stepped block; (**c**) the wrapped phase and fringe order of the stepped block; (**d**) the unwrapped phase of the stepped block; (**e**) the reconstructed 3D result of the stepped block; (**f**) fitting deviation of plane $\Pi_{A}$; (**g**) fitting deviation of plane $\Pi_{B}$.

Accuracy verification was performed using a stepped block consisting of two blocks, A and B, as shown in Figure 10a. The absolute error of plane height difference $\varepsilon_{\mathrm{height}\;}=\left|H_{m}-H_{r}\right|$ and the plane fitting standard deviation $\varepsilon_{std}=\sqrt{\sum_{i=1}^{n}{\left(dis_{i}\right)}^{2}/n}$ (including $\varepsilon_{std}^{A}$ of plane $\Pi_{A}$ and $\varepsilon_{std}^{B}$ of plane $\Pi_{B}$) were used as evaluation indicators, where $H_{r}$ and $H_{m}$ are the true height difference (8.874 mm) and the measured height difference between $\Pi_{A}$ and $\Pi_{B}$, respectively, and $dis_{i}$ is the distance from the *i*-th point to the fitting plane.

A fringe image of the stepped block captured under normal exposure conditions is shown in Figure 10b, the phase calculation results are shown in Figure 10c,d, and the reconstructed point cloud is shown in Figure 10e. The point clouds of $\Pi_{A}$ and $\Pi_{B}$ were selected for plane fitting, and the fitting deviations are shown in Figure 10f,g.

The measured data of the stepped block are shown in Table 3. It can be seen that the proposed method is superior to traditional SPU (fringe frequency is 30), which is because we increased the fringe frequency (fringe frequency is 73); it is comparable to Jiang’s time-domain algorithm in accuracy because both are based on a phase-shifting algorithm.

**Figure 11:**
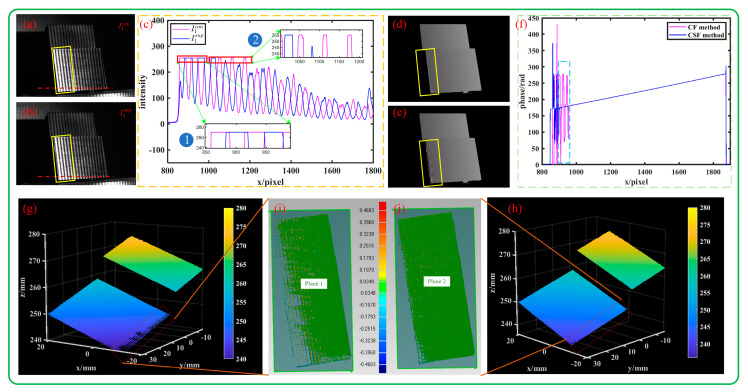
Stepped block measurement under overexposure. (**a**,**b**) The fringe patterns of the stepped block; (**c**) comparison of grayscale distribution of a certain row; (**d**,**e**) the calculated phases; (**f**) comparison of phase distribution of a certain row; (**g**,**h**) the reconstructed 3D result; (**i**,**j**) the point cloud fitting deviation of plane 1 and plane 2 in overexposed areas. Note: the yellow boxes in (**a**,**b**,**d**,**e**) indicate the overexposed areas.

The conventional and supplemental pattern images of the stepped block captured under overexposure conditions are shown in Figure 11a,b, the grayscale distribution of a certain row is shown in Figure 11c, the phase calculation results are shown in Figure 11d,e, the phase comparison curve is shown in Figure 11f, and the reconstructed point cloud is shown in Figure 11g,h. The point cloud fitting deviations in overexposed areas are shown in Figure 11i,j, and the plane fitting standard deviations are 0.018 mm and 0.035 mm, respectively.

In the partial enlargement area ① of Figure 11c, both the conventional fringes and the supplemental fringes are overexposed; in the partial enlargement area ②, the conventional fringes are overexposed, but the supplemental fringes are not overexposed. From the phase results in Figure 11c,d, we can see that the phase at the overexposed point is smoother when using conventional and supplemental fringes than when using only conventional fringes. From Figure 11e, we can see that although there are jumps, the jump range and number of jumps using the CSF method are far lower than those using the CF method. In addition, it can be seen from the reconstruction results in Figure 11g,h and the fitting deviation in Figure 11i,j that in these two overexposure situations, the CSF method can reduce the reconstruction error to a certain extent. The specific performance is as follows: the completeness of the CSF method is lower than that of the CF method, and the flatness is worse. Therefore, the introduction of supplemental fringes can reduce the reconstruction error caused by the saturation of conventional fringes.

The measured data of the stepped block under overexposure are shown in Table 4. Since the error mainly comes from the wrapping phase, the CSF method and Jiang’s algorithm also have considerable accuracy, higher than that of the CF method, in the case of overexposure.

#### 3.2.2. Isolated Object Measurement

**Figure 12:**
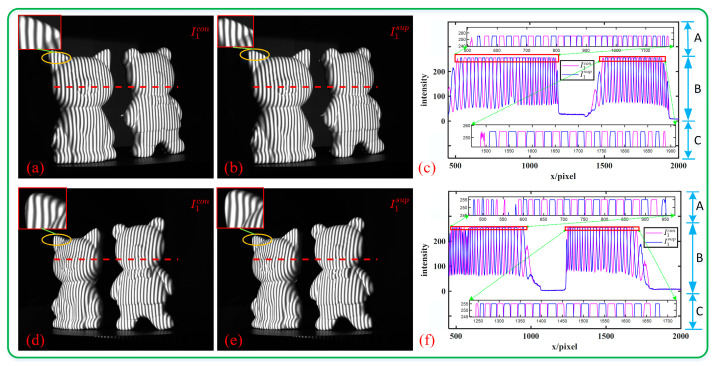
Schematic diagram of the overexposed area. (**a**,**d**) $I_{1}^{con}(x,y)$ of C_main_ and C_aux_; (**b**,**e**) $I_{1}^{sup}(x,y)$ of C_aux_. The red rectangular area in the upper left corner is a partial enlargement of the overexposed position. (**c**,**f**) Grayscale distribution maps of a certain row of C_main_ and C_aux_, where area B is the original distribution and areas A and C are partial enlargements at overexposed pixels.

We performed a 3D reconstruction of two separated plaster statues to confirm the effectiveness of the proposed algorithm on isolated objects. Figure 12a,d, and Figure 12b,e show two of the fringe patterns of C_main_ and C_aux_, i.e., $I_{1}^{con}(x,y)$ and $I_{1}^{sup}(x,y)$ in Equations (8) and (10). The extracted cross-sections of the fringes are shown in Figure 12c,f. It can be seen that if the pixels in $I_{1}^{con}(x,y)$ are saturated, the pixels in $I_{1}^{sup}(x,y)$ are not saturated. Therefore, when calculating the phase, we can use $I_{1}^{sup}(x,y)$ instead of $I_{1}^{con}(x,y)$ to avoid phase errors.

**Figure 13:**
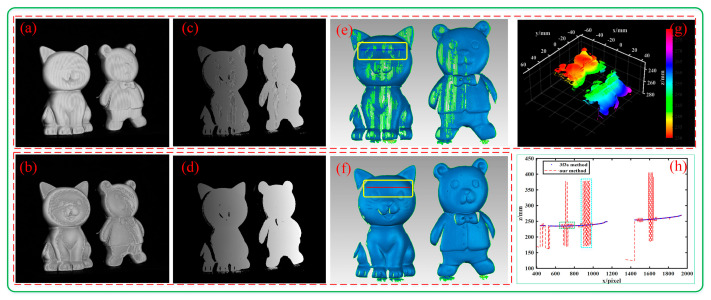
Results of isolated objects: (**a**,**b**) modulation using the CF method and the CSF method, respectively; (**c**,**d**) phases using the CF method and the CF method, respectively; (**e**,**f**) reconstructed surfaces using the CF method and the CSF method, respectively; (**g**) reconstructed point cloud using the CF method; (**h**) z-value distribution curve along a certain row using the CF method and the CSF method.

Figure 13a–f show the modulation, phase, and 3D surface reconstruction results with the CSF method and the CF method. Obviously, Figure 13a,c have uneven modulation and phase caused by high fringe intensity saturation, which in turn leads to ripples and large missing areas on the 3D reconstructed surface in Figure 13e. In contrast, the modulation in the overexposure area of Figure 13b is relatively smooth, and the phase of Figure 13d is overall smooth, so the 3D reconstruction result in Figure 13f is complete and the local details are also clear.

To better illustrate the measurement effect, we plotted point clouds with the CF method, as shown in Figure 13g. It can be seen that the reconstructed point cloud will shift significantly at overexposed locations. At the same time, we drew the z-value distribution of the blue line and red line in Figure 13c,f, as shown in Figure 13h. It can be seen that the z-value deviation in the dotted-line box on the left is not particularly large, which corresponds to ripples on the surface of the object, while the z-value deviation in the dotted-line box on the right is particularly large, which corresponds to missing parts on the surface of the object, because these positions will not be considered when surfacing the point cloud. Therefore, the proposed method can be significantly adapted to reduce saturation-induced phase errors of isolated objects.

#### 3.2.3. Measurement Completeness

To verify the measurement completeness performance of the proposed method, the proposed method and the multi-exposure fusion method [9] were used to measure an aero-engine turbine blade.

**Figure 14:**
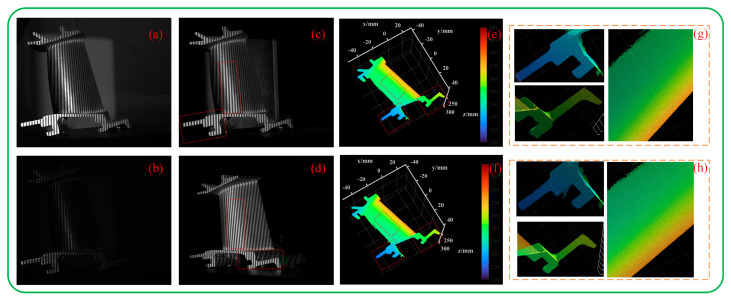
Multi-exposure images and reconstruction results: (**a**) high-exposure image of the left camera; (**b**) low-exposure image of the left camera; (**c**,**d**) fusion images of the left camera and right camera; (**e**) the 3D point cloud reconstructed by the multi-exposure fusion method [9]; (**f**) the 3D point cloud reconstructed by the proposed method; (**g**) a partial enlarged view of (**e**); (**h**) a partial enlarged view of (**f**). Note: the red boxes represent the overexposed areas.

Image fusion was performed based on nine groups of images. The high- and low-grayscale images of the left camera are shown in Figure 14a,b. The images were fused according to different exposures, and the generated fused images are shown in Figure 14c,d. The point cloud obtained by 3D reconstruction based on the fused image is shown in Figure 14e, and the point cloud obtained based on the proposed algorithm is shown in Figure 14f. Their partial enlarged views are shown in Figure 14g,h, respectively. From these views, it can be seen that the completeness of this method is close to that of the nine-exposure fusion method. But the proposed method only uses eight fringes, and the number of fringes using the nine-exposure technique is 9 × 4 = 36. Therefore, the measurement efficiency of the proposed method is 4.5 times higher than that of the nine-exposure technique.

#### 3.2.4. Measurement Flexibility

**Figure 15:**
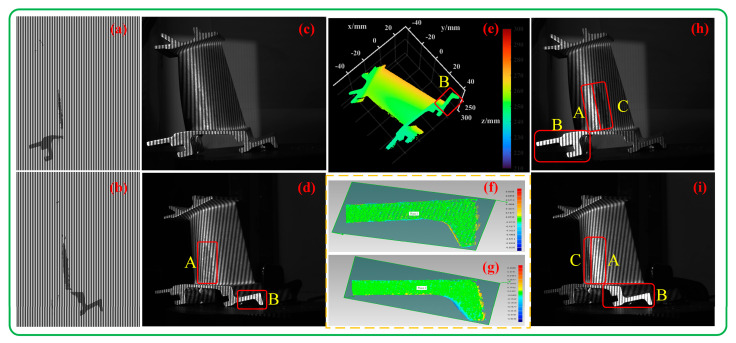
Adaptive images and results: (**a**) adaptive patterns of the left camera; (**b**) adaptive patterns of the right camera; (**c**) left image captured using the adaptive patterns of the left camera; (**d**) right image captured using the adaptive patterns of the right camera; (**e**) the reconstructed 3D point cloud; (**f**) error distribution diagram of plane (plane 1) fitting for the B region of (**e**) using the proposed method; (**g**) error distribution diagram of plane (plane 2) fitting for the B region of (**e**) using the adaptive projection method [15]; (**h**) left image captured using the adaptive patterns of the right camera; (**i**) right image captured using the adaptive patterns of the left camera.

To verify the measurement flexibility performance of the proposed method, the aero-engine turbine blade was further measured with the adaptive projection method [15]. The generated adaptive fringe images are shown in Figure 15a,b. It can be seen that the grayscale of the pattern corresponding to the highly reflective area in the image is uniformly reduced. Finally, the resulting adaptive fringe image was projected to complete the measurement.

The captured images are shown in Figure 15c,d, where the original highly reflective area is under the projection of the new fringes, and the saturation phenomenon is obviously weakened, as shown in areas A and B in Figure 15c and area A in Figure 15d. However, since the projected image reduces the gray level as a whole, it is not adjusted for the reflection of each point, and the highest projection gray level is set based on empirical values, so in the newly captured image, some areas may still be overexposed, as shown in area B in Figure 15d. The 3D reconstruction result shown in Figure 15e was obtained.

In order to quantitatively evaluate the reconstruction effect of the overexposed area B, we use the method presented in [15] and the proposed method to reconstruct the point cloud in this area and perform plane fitting (plane 1 and plane 2, respectively). The obtained error distribution is shown in Figure 15f,g. It can be seen that plane 1 has ripples, while plane 2 is relatively smooth. The calculated standard deviations are 0.041 mm and 0.085 mm, respectively, which shows that the accuracy of the proposed method is higher than that of the method presented in [15].

If we change the angle of view, here, for the sake of simplicity, it is assumed that the adaptive patterns generated for the right image are projected and captured by the left camera, as shown in Figure 15h. The saturation in areas A and B in Figure 15h is not removed at this time. It just makes the grayscale of area C that is not originally exposed become lower. There is also a similar situation in Figure 15i. This is easy to understand because the adaptive adjustment is based on the precise correspondence between the overexposed area of the object and the pixels of the projector. If the viewing angle is changed, this correspondence will be broken, so the measurement of the reflective area will be invalid. Our method is not subject to this limitation, making it more flexible than adaptive projection methods.

## 4. Conclusions

We propose a phase retrieval method for HDR 3D measurement: Four conventional patterns are used for phase recovery under normal exposure conditions, and corresponding π phase-shifting supplementary patterns are used for phase recovery under overexposure conditions. Our method can reduce the phase error in the overexposed area of the object being measured and does not require changing the camera exposure time or adaptively generating fringes based on the surface of the object. The experimental results verified its feasibility. The main value of this method is reflected in the following two aspects:

(1) The fringe frequency of SPU is extended to improve measurement accuracy while inheriting the high measurement efficiency of the method. For binocular systems, several other techniques [36,37] can decrease the count of fringe patterns. Yet, they typically rely on complex, time-intensive spatial domain computations or embedding pattern methods. In contrast, our method is computationally simpler and easier to implement.
(2) Taking advantage of stereo cameras, phase recovery can be achieved by projecting an additional image on the basis of three-step phase shifting. There are also other methods that can perform phase unwrapping using only three or four fringes, but using π phase-shifting fringes to suppress the phase error in the overexposed area often leads to phase recovery failure, as seen in [38,39]. We only need to add four additional corresponding π phase-shifting fringes to deal with different overexposure situations. We reduced the number of fringes to 4/10 that of Jiang’s method. In addition, modulation calculation (for background removal) is added, and the dynamic range improvement capability is simply quantitatively analyzed (the dynamic range of the FPP system can be increased to twice that of the traditional method).

In future work, there are still the following aspects worthy of continuous improvement:

(1) Since our method has good flexibility and uses a smaller number of patterns, the integration of real-time measurements into existing frameworks can be considered later.
(2) Our method only amplifies the dynamic range twofold, so it is necessary to further expand the 3D measurement’s dynamic range.

## Figures and Tables

**Table 1:** Different exposure cases.

Case	Overexposed Pattern(s)	Used Patterns
1: No overexposed pattern	1_1: None	$I_{1}^{con},I_{2}^{con},I_{3}^{con},I_{4}^{con}$
2: An overexposed pattern	2_1: $I_{1}^{con}$	$I_{1}^{sup},I_{2}^{con},I_{3}^{con},I_{4}^{con}$
	2_2: $I_{2}^{con}$	$I_{1}^{con},I_{2}^{sup},I_{3}^{con},I_{4}^{con}$
	2_3: $I_{3}^{con}$	$I_{1}^{con},I_{2}^{con},I_{3}^{sup},I_{4}^{con}$
	2_4: $I_{4}^{con}$	$I_{1}^{con},I_{2}^{con},I_{3}^{con},I_{4}^{sup}$
3: Two overexposed patterns	3_1: $I_{1}^{con},I_{2}^{con}$	$I_{1}^{sup},I_{2}^{sup},I_{3}^{con},I_{4}^{con}$
	3_2: $I_{1}^{con},I_{3}^{con}$	$I_{1}^{sup},I_{2}^{con},I_{3}^{sup},I_{4}^{con}$
	3_3: $I_{1}^{con},I_{4}^{con}$	$I_{1}^{sup},I_{2}^{con},I_{3}^{con},I_{4}^{sup}$
	3_4: $I_{2}^{con},I_{3}^{con}$	$I_{1}^{con},I_{2}^{sup},I_{3}^{sup},I_{4}^{con}$
	3_5: $I_{2}^{con},I_{4}^{con}$	$I_{1}^{con},I_{2}^{sup},I_{3}^{con},I_{4}^{sup}$
	3_6: $I_{3}^{con},I_{4}^{con}$	$I_{1}^{con},I_{2}^{con},I_{3}^{sup},I_{4}^{sup}$
4: Three overexposed patterns	4_1: $I_{1}^{con},I_{2}^{con},I_{3}^{con}$	$I_{1}^{sup},I_{2}^{sup},I_{3}^{sup},I_{4}^{con}$
	4_2: $I_{1}^{con},I_{2}^{con},I_{4}^{con}$	$I_{1}^{sup},I_{2}^{sup},I_{3}^{con},I_{4}^{sup}$
	4_3: $I_{1}^{con},I_{3}^{con},I_{4}^{con}$	$I_{1}^{sup},I_{2}^{con},I_{3}^{sup},I_{4}^{sup}$
	4_4: $I_{2}^{con},I_{3}^{con},I_{4}^{con}$	$I_{1}^{con},I_{2}^{sup},I_{3}^{sup},I_{4}^{sup}$
5: Four overexposed patterns	5_1: $I_{1}^{con},I_{2}^{con},I_{3}^{con},I_{4}^{con}$	$I_{1}^{sup},I_{2}^{sup},I_{3}^{sup},I_{4}^{sup}$

**Table 2:** Phase unwrapping formula.

Case	Formula
2_1	$\;\frac{\sqrt{3}(3I_{1}-2I_{2}-I_{3})}{3}sinC_{s}+(I_{3}-I_{1})cosC_{s}+(I_{1}-I_{2}-I_{3})+I_{4}=0$
2_2	$\;\frac{\sqrt{3}(I_{3}-I_{1})}{3}sinC_{s}+(2I_{2}-I_{1}-I_{3})cosC_{s}+(I_{2}-I_{1}-I_{3})+I_{4}=0$
2_3	$\;\frac{\sqrt{3}(I_{1}+2I_{2}-3I_{3})}{3}sinC_{s}+(I_{1}-I_{3})cosC_{s}+(I_{3}-I_{2}-I_{1})+I_{4}=0$
2_4	$\;\frac{\sqrt{3}(I_{1}-I_{3})}{3}sinC_{s}+\frac{\left(2I_{2}-I_{1}-I_{3}\right)}{3}cosC_{s}-\frac{\left(I_{1}+I_{2}+I_{3}\right)}{3}+I_{4}=0$
3_1	$\;\frac{\sqrt{3}(3I_{3}-2I_{2}-I_{1})}{3}sinC_{s}+(I_{3}-I_{1})cosC_{s}+(I_{3}-I_{2}-I_{1})+I_{4}=0$
3_2	$\;\frac{\sqrt{3}(I_{1}-I_{3})}{3}sinC_{s}+(I_{1}-2I_{2}+I_{3})cosC_{s}+(I_{2}-I_{1}-I_{3})+I_{4}=0$
3_3	$\;\frac{\sqrt{3}(2I_{2}-3I_{1}+I_{3})}{3}sinC_{s}+(I_{1}-I_{3})cosC_{s}+(I_{1}-I_{2}-I_{3})+I_{4}=0$
3_4	$\;\frac{\sqrt{3}(2I_{2}-3I_{1}+I_{3})}{3}sinC_{s}+(I_{1}-I_{3})cosC_{s}+(I_{1}-I_{2}-I_{3})+I_{4}=0$
3_5	$\;\frac{\sqrt{3}(I_{1}-I_{3})}{3}sinC_{s}+(I_{1}-2I_{2}+I_{3})cosC_{s}+(I_{2}-I_{1}-I_{3})+I_{4}=0$
3_6	$\;\frac{\sqrt{3}(3I_{3}-2I_{2}-I_{1})}{3}sinC_{s}+(I_{3}-I_{1})cosC_{s}+(I_{3}-I_{2}-I_{1})+I_{4}=0$
4_1	$\;\frac{\sqrt{3}(I_{1}-I_{3})}{3}sinC_{s}+\frac{\left(2I_{2}-I_{1}-I_{3}\right)}{3}cosC_{s}-\frac{\left(I_{1}+I_{2}+I_{3}\right)}{3}+I_{4}=0$
4_2	$\;\frac{\sqrt{3}(I_{3}+2I_{2}-3I_{1})}{3}sinC_{s}+(I_{1}-I_{3})cosC_{s}+(I_{3}-I_{2}-I_{1})+I_{4}=0$
4_3	$\;\frac{\sqrt{3}(I_{3}-I_{1})}{3}sinC_{s}+\frac{\left(2I_{2}-I_{1}-I_{3}\right)}{3}cosC_{s}+(I_{2}-I_{1}-I_{3})+I_{4}=0$
4_4	$\;\frac{\sqrt{3}(3I_{1}-2I_{2}-I_{3})}{3}sinC_{s}+(I_{3}-I_{1})cosC_{s}+(I_{1}-I_{2}-I_{3})+I_{4}=0$
5_1	$\;\frac{\sqrt{3}(I_{3}-I_{1})}{3}sinC_{s}+\frac{\left(I_{1}-2I_{2}+I_{3}\right)}{3}cosC_{s}-\frac{\left(I_{1}+I_{2}+I_{3}\right)}{3}+I_{4}=0$

**Table 3:** Error statistics under normal exposure (units: mm).

	SPU Method	Jiang’s Method [28]	CF Method
$\varepsilon_{height}$	0.0397	0.0258	0.0259
$\varepsilon_{std}^{A}$	0.0194	0.0143	0.0150
$\varepsilon_{std}^{B}$	0.0241	0.0212	0.0203

**Table 4:** Error statistics under overexposure (units: mm).

	Jiang’s Method [28]	CF Method	CSF Method
$\varepsilon_{height}$	0.0269	0.0405	0.0271
$\varepsilon_{std}^{A}$	0.0164	0.0266	0.0172
$\varepsilon_{std}^{B}$	0.0209	0.0204	0.0201

## Data Availability

Not applicable.
